# A rare case of pneumorrhachis accompanying spontaneous pneumomediastinum

**DOI:** 10.1590/0100-3984.2015.0031

**Published:** 2017

**Authors:** Luciana Mendes Araújo Borem, Dimitrius Nikolaos Jaconi Stamoulis, Ana Flávia Mundim Ramos

**Affiliations:** 1 Santa Casa de Montes Claros, Montes Claros, MG, Brazil.; 2 Universidade Federal do Triângulo Mineiro (UFTM), Uberaba, MG, Brazil.

**Figure 1:**
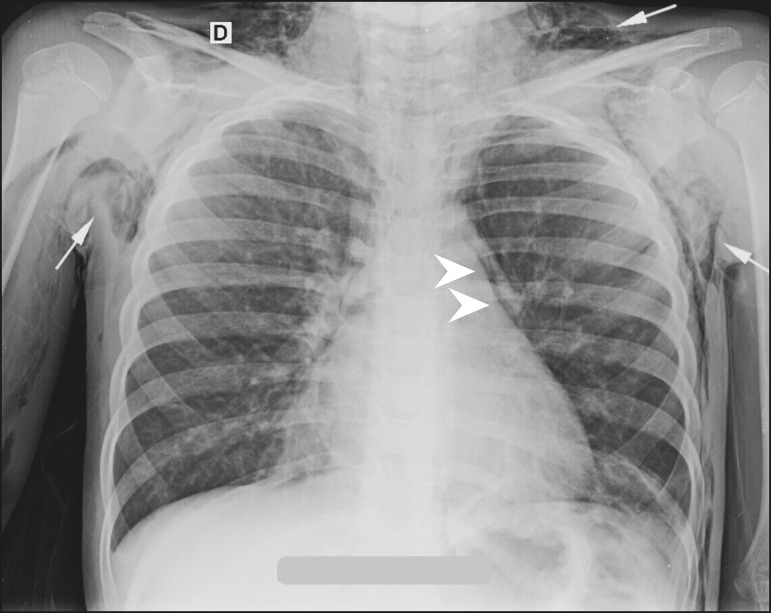
Posteroanterior chest X-ray showing pneumomediastinum (arrowheads), together with extensive subcutaneous emphysema in the supraclavicular and axillary regions (arrows).

**Figure 2:**
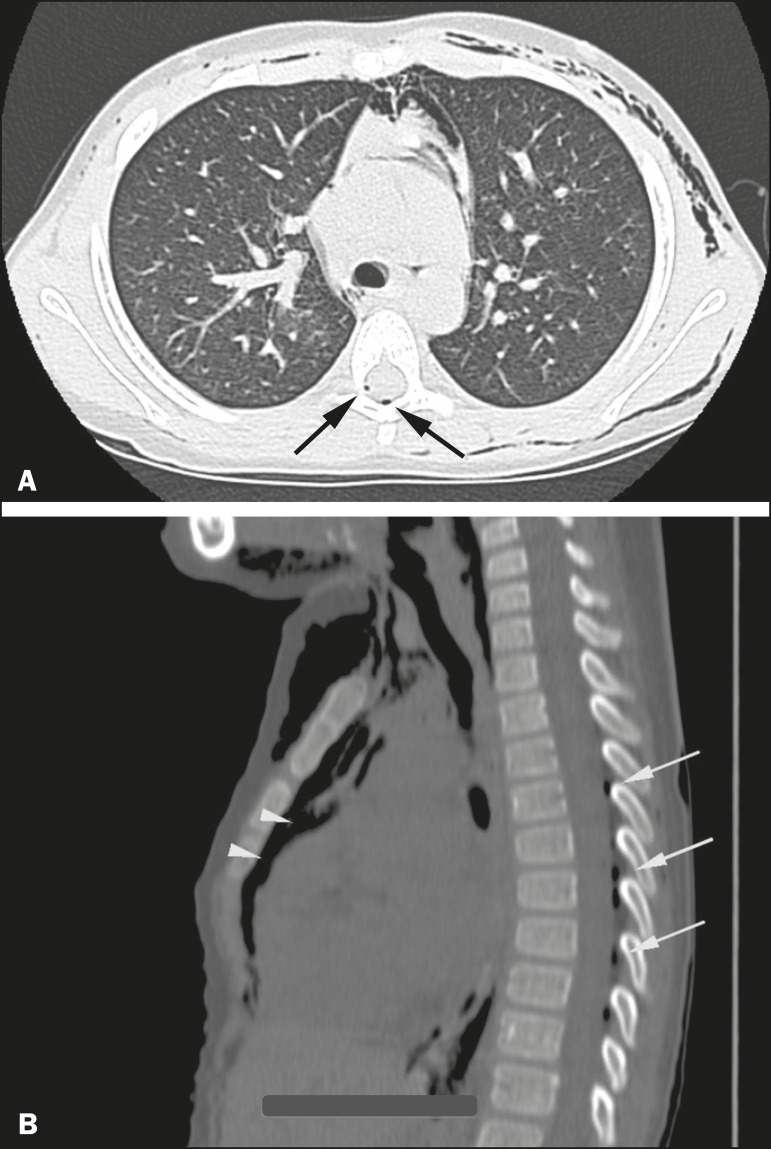
CT of the chest in the axial (A) and sagittal (B) planes showing pneumorrhachis (arrows) and mediastinal emphysema (arrowheads).

Dear Editor,

A 7-year-old female with dyspnea and edema of the neck, accompanied by a cough, was treated at another facility, where anti-inflammatory drugs and an inhaler were prescribed. The patient evolved to worsening of the dyspnea and cough, in addition to intercostal retraction and increased neck volume. She presented to our facility in satisfactory general health. On physical examination, the oropharynx showed no alterations, although there was bilateral edema of the neck and periorbital area, together with diminished breath sounds, sparse wheezing, respiratory rate of 30 breaths/min, intercostal retraction, and subcutaneous crackles on anterior/posterior thoracic palpation, without Hamman’s sign. A chest X-ray obtained at admission (Figure 1) showed pneumomediastinum and extensive subcutaneous emphysema. She underwent computed tomography (CT) of the chest (Figure 2), which revealed pneumorrhachis, a rare finding. The patient remained in the hospital for five days under supportive care, and there was complete remission of symptoms.

Spontaneous pneumomediastinum, also known as Hamman’s syndrome, is an uncommon condition in medical practice, occurring in approximately 1/30,000 hospital admissions^(1)^ and in only 1% of asthma cases^(2)^. Its main causes are intense physical exercise, labor (of childbirth), pulmonary barotrauma, diving to great depths, severe paroxysmal coughing, vomiting, asthma, inhalation of narcotics, bronchial asthma, and a slender body type^(1,2)^. 

The pathophysiological hallmark of Hamman’s syndrome is alveolar overdistension and rupture, which results from high intra-alveolar pressure, low perivascular pressure, or both. After the initial event, the air freely penetrates the mediastinum during the respiratory cycle, in order to balance the pressure gradients^(3,4)^. Known triggers include acute exacerbation of asthma and situations requiring the Valsava maneuver^(4)^.

The combination of spontaneous pneumomediastinum and pneumorrhachis is extremely rare^(5,6)^. Possible causes of pneumorrhachis include use of the drug ecstasy, abscesses, asthma attacks, coughing fits, violent vomiting, epidural anesthesia, lumbar puncture, and thoracic or vertebral surgery or trauma^(7,8)^. In extremely rare cases, meningitis or pneumocephalus can occur^(7)^. Pneumorrhachis typically occurs directly, when atmospheric air reaches the epidural space by means of a needle or a penetrating wound from the spine, although it can occur indirectly, as in bronchial asthma. In the case of bronchial asthma, air from the rupture of a peripheral pulmonary alveolus leaks into the pulmonary perivascular interstitium and follows the path of least resistance of the mediastinum to the fascia of the neck. Due to the absence of fascial barriers, air crosses the neural foramen and deposits in the epidural space. In either situation, pneumorrhachis is usually asymptomatic and disappears spontaneously within a few weeks.

Whereas CT allows direct visualization of the presence of air in the affected compartment(s), X-rays can reveal signs typical of pneumomediastinum, produced when the air leaving the mediastinum delineates the normal anatomical structures. Such signs include subcutaneous emphysema, the “sail sign” of the thymus, pneumopericardium, the “ring-around-the-artery” sign, the “continuous diaphragm” sign, and the “double bronchial wall” sign^(3)^.
